# The Kinetics of Early T and B Cell Immune Recovery after Bone Marrow Transplantation in RAG-2-Deficient SCID Patients

**DOI:** 10.1371/journal.pone.0030494

**Published:** 2012-01-25

**Authors:** Atar Lev, Amos J. Simon, Mor Bareket, Bella Bielorai, Daphna Hutt, Ninette Amariglio, Gideon Rechavi, Raz Somech

**Affiliations:** 1 Cancer Research Center, Tel Hashomer, Israel, affiliated to the Sackler Faculty of Medicine, Tel Aviv University, Tel Aviv, Israel; 2 Pediatric Immunology Service, Jeffery Modell Foundation (JMF) Center, Tel Hashomer, Israel, affiliated to the Sackler Faculty of Medicine, Tel Aviv University, Tel Aviv, Israel; 3 Pediatric Hematology/Oncology Division and Bone Marrow Transplantation Unit, Safra Children's Hospital, Sheba Medical Center, Tel Hashomer, Israel, affiliated to the Sackler Faculty of Medicine, Tel Aviv University, Tel Aviv, Israel; 4 Hematology Laboratory, Sheba Medical Center, Tel Hashomer, Israel, affiliated to the Sackler Faculty of Medicine, Tel Aviv University, Tel Aviv, Israel; Albert Einstein College of Medicine, United States of America

## Abstract

The kinetics of T and B cell immune recovery after bone marrow transplantation (BMT) is affected by many pre- and post-transplant factors. Because of the profoundly depleted baseline T and B cell immunity in recombination activating gene 2 (RAG-2)-deficient severe combined immunodeficiency (SCID) patients, some of these factors are eliminated, and the immune recovery after BMT can then be clearly assessed. This process was followed in ten SCID patients in parallel to their associated transplant-related complications. Early peripheral presence of T and B cells was observed in 8 and 4 patients, respectively. The latter correlated with pre-transplant conditioning therapy. Cells from these patients carried mainly signal joint DNA episomes, indicative of newly derived B and T cells. They were present before the normalization of the T cell receptor (TCR) and the B cell receptor (BCR) repertoire. Early presentation of the ordered TCR gene rearrangements after BMT occurred simultaneously, but this pattern was heterogeneous over time, suggesting different and individual thymic recovery processes. Our findings early after transplant could suggest the long-term patients' clinical outcome. Early peripheral presence of newly produced B and T lymphocytes from their production and maturation sites after BMT suggests donor stem cell origin rather than peripheral expansion, and is indicative of successful outcome. Peripheral detection of TCR excision circles and kappa-deleting recombination excision circles in RAG-2-deficient SCID post-BMT are early markers of T and B cell reconstitution, and can be used to monitor outcome and tailor specific therapy for patients undergoing BMT.

## Introduction

Severe combined immunodeficiency (SCID) is characterized by significantly low levels of T and B cells and profound defective immune function. Bone marrow transplantation (BMT) is the life-saving and life-sustaining treatment procedure for such patients in order to restore their T and B cell immunity [1]. After BMT, the three main goals that are extremely important for achieving long-term survival in these patients include engraftment of the transfused stem cells, prevention of graft versus host disease (GVHD) and neogenesis of functionally diverse and matured T and B cells [2]. The kinetics of early T and B cell recovery after BMT, occurring during the first three months post-BMT, has a major impact on achieving these goals.

The thymus and the bone marrow are the primary anatomic sites for T and B cell neogenesis from undifferentiated hematopoietic progenitor cells. Within these organs, hematopoietic progenitor cells that have been committed to the T and B cell lineage undergo rapid proliferation and differentiation to mature cells. During this process, a diverse receptor repertoire is formed, and the resulting cells are able to respond to a wide array of internally and externally processed antigens [3]–[5]. Normally, T cell maturation in the thymus progresses through distinct stages which are defined phenotypically by the expression of the T cell receptor (TCR) and the CD4 and CD8 co-receptors. On the basis of the expression of these cell surface markers and the ordered gene rearrangements, thymocytes represent different maturation steps on their way to becoming mature cells [6]. On the one hand, DNA strand breakage during the thymic and bone marrow maturation processes of the TCR α/β chains and the B cell receptor (BCR) light and heavy chains, respectively, creates functional receptors (i.e., the formation of coding joint recombination sites), while, on the other hand, it creates byproducts (i.e., the formation of signal joint recombination sites) termed TCR excision circles (TRECs) and kappa-deleting recombination excision circles (KRECs), respectively [7]. TREC quantification is extensively used as an accurate measure of thymic function and T cell neogenesis, and this analysis was therefore suggested as a diagnostic tool for T cell immunodeficiency [8], for neonatal screen assay to detect SCID immediately after birth [9], and as being the most predictive factor for long-term T cell immune reconstitution after BMT [10]. KRECs form the extra-chromosomal (episomal) excision product of the immunoglobulin gene rearrangement. Similar to TRECs, these episomal products cannot replicate in the cell. KRECs appear to be highly stable structures, which can persist for a considerable length of time in peripheral blood. The ratio between genomic coding joints and signal joints on these circles reflects both B cell neogenesis and the replication history of B lymphocyte subsets. As such, KRECs can be found not only in precursor B cells but in mature B lymphocytes as well [11]. After BMT, the detection of KRECs reflects newly derived functional bone marrow B cells.

A major challenge in the field of BMT is the overcoming the difficulty of monitoring the efficacy of the procedure, especially since the prognosis is affected by many pre- and post-transplant parameters [1]. Ideally, T and B cells should regenerate from stem cells present in the graft, and various BMT procedure manipulations, such as pre-transplant conditioning therapy, are performed in order to ensure its occurrence. Recombination activating genes 1 and 2 (RAG-1 and RAG-2) are the two essential and tissue-specific components for the generation of antigen-binding diversity by assembly of the variable (V), diversity (D), and joining (J) DNA segments at both the immunoglobulin and TCR gene loci during lymphocyte development. Therefore, assessment of T and B cell regeneration can be clearly demonstrated in SCID patients with RAG-1 or RAG-2 defects after BMT. Unlike other SCID variants, RAG-deficient patients suffer from low numbers of T and B cells, and the cells that they do have are dysfunctional. Thus, the detection of T and B cell undergoing neogenesis and the measurement of a broad formed repertoire in RAG-deficient SCID patients depend primarily on the differentiation of cells originating from donor undifferentiated hematopoietic progenitor cells and not from the host cells.

In the present study, we examined the early release of newly formed T and B cells from the thymus and the bone marrow in the peripheral blood of 10 RAG-2-deficient SCID patients who received a transplantation of hematopoietic stem cells from healthy donors. This experimental population provides a unique opportunity to examine immune reconstitution and to identify clinical factors that influence the rapidity and extent of T and B cell neogenesis in vivo.

## Methods

### Patients

Ten consecutive patients diagnosed at the Sheba Medical Center (Tel Hashomer, Israel) as having RAG-2-deficient SCID were enrolled in this study which was approved by the Institutional Review Board. Parents provided signed informed consent to obtain blood from their children. Diagnosis was based on clinical findings, family history, and immunological evaluation, and confirmed by genetic testing. Patients who lacked histocompatible siblings or closely matched related donors (MRD) received match unrelated donor (MUD) or mismatch related donor (MMRD) BMTs. The minimum follow-up after BMT was 6 months. Lymphocytes in all patients were tested for human leukocyte antigen (HLA) A, B, C and DR typing using standard serological methods, or DNA hybridization with sequence-specific oligonucleotide probes in order to find a suitable donor.

### Transplantation procedures

The patients were treated in positive pressure with heap filter units from the time of diagnosis until discharge. Trimethoprim/sulfametaxozole was used for Pneumocystis jiroveci prophylaxis. Intravenous immunoglobulins (IVIG, 600 mg/kg) were infused to maintain IgG levels above 6 g/l. Acyclovir (1500 mg/m^2^/day) was administered to patients who received bone marrow from cytomegalovirus (CMV)-seropositive donors or to CMV-seropositive patients who received stem cell transplantation from a seronegative donor. All blood products were CMV safe and irradiated. Pre-transplant conditioning therapy consisting of various drugs was given to some patients, and the severity of GVHD was graded as described previously [10]. It was defined as being chronic if it presented or occurred more than 100 days after transplant, even though the symptoms were often similar to acute GVHD. GVHD prophylaxis, administered to some patients from the day prior to transplant, consisted of cyclosporin A combined with methotrexate. All patients were infused with non-depleted 3–5×108 donor bone marrow mononuclear cells per kg of recipient weight. They received granulocyte colony stimulating factor (G-CSF) from the day of transplant until the absolute neutrophil count was >1.0×10^9^/l for 3 consecutive days.

### Engraftment and chimerism studies

Neutrophil engraftment was considered to have occurred on the first of 3 consecutive days in which the absolute neutrophil count exceeded 0.5×10^9^/l. Lymphocytes were analyzed for long-term donor engraftment by polymerase chain reaction (PCR) of polymorphic markers. Multiplex tetranucleotide repeat PCR was performed on 2 ng of genomic DNA using the AmpFlSTR SGM Plus kit (Applied Biosystems), which amplifies 10 tetranucleotide repeat loci (plus the X–Y homologous gene amelogenin) labeled in three different colors (blue 5-FAM, green JOE and yellow NED). Amplified PCR products were subjected to capillary electrophoresis in an ABI Prism 3100 (Applied Biosystems) automated DNA sequencer under the conditions recommended by the manufacturer. The GeneScan 3.7 Analysis software (Applied Biosystems) was used for the analysis of the PCR products.

### Lymphocyte markers and T-cell proliferative responses

Cell surface markers of peripheral blood mononuclear cells (PBMCs), using immunofluorescent staining and flow cytometry (Epics V; Coulter Electronics, Hialeah, FL) with antibodies purchased from Coulter Diagnostics, and lymphocyte proliferation in response to phytohemagglutinin, using tritiated thymidine incorporation, were determined as previously described [12]. For lymphocyte proliferation studies, cells were harvested 3 days after transplantation and samples were counted in a liquid scintillation counter. All assays were performed in triplicate, and a stimulation index (SI) was calculated as the ratio between stimulated and unstimulated lymphocyte responses and compared with the SI obtained from normal controls. Serum concentrations of immunoglobulins were measured using nephelometry.

### BCR and TCR gene rearrangement analyses

TCR and BCR gene rearrangement analyses were performed on genomic DNA obtained from patient's PBMCs without specific cell enrichment, as previously described.^8^ Briefly, the immunoglobulin heavy chain (IGH) and TCR-γ genes were amplified by PCR using fluorescence-labeled Vh primers or Vγ primers, respectively, according to the standardized Biomed 2 protocol. Fluorescence-labeled PCR products (1 µl of each) were added to a mixture of 8.5 µl deionized formamide and 0.5 µl GeneScan 500TM Rox internal lane standard (PE Applied Biosystem, Germany), and separated using the 3100 Genetic Analyzer (Applied Biosystem). Results were analyzed using the GeneMapper software (Applied Biosystem).

### Quantification of TRECs and KRECs

The amount of TRECs was determined by real-time quantitative (RQ)-PCR. Reactions were performed using 0.25 µg genomic DNA extracted from the patient's PBMCs. PCR reactions contained TaqMan universal PCR master mix (Applied Biosystems,), specific primers (900 nM) and FAM-TAMRA probes (250 nM) as previously described [8] (Table 1). RQ-PCR was carried out in an ABI PRISM 7900 Sequence Detector System (Applied Biosystems). The standard curve was constructed by using serial dilutions of a known TREC plasmid (generously provided by Dr. Daniel Douek, Vaccine Research Center, National Institute of Allergy and Infectious Diseases, Bethesda, MD). The dilutions contained 10^3^ to 10^6^ copies of the signal joint TREC plasmid. A triplicate was used for each dilution. The obtained cycle threshold (Ct) values of the standard curve were the same in all experiments ±0.5 cycle: 20.8 cycles for 10^6^ copies, 23.6 cycles for 10^5^ copies, 27.7 cycles for 10^4^ copies, and 30.1 cycles for 10^3^ copies. TREC copies were >400 in 40 samples in which immunodeficiency was ruled out. The number of TRECs in a given sample was automatically calculated by comparing the obtained Ct value of a patient's sample to the standard curve using an absolute quantification algorithm.

**Table 1:** List of primers used by real time PCR analysis to detect the amount of TRECs, signal joint KRECs, coding joint KRECs and the various TCR rearrangements.

	Forward primer (5′-3′)	Reverse primer (5′-3′)	Taqman probe (FAM-TAMRA) (5′-3′)
TREC	CACATCCCTTTCAACCATGCT	GCCAGCTGCAGGGTTTAGG	ACACCTCTGGTTTTTGTAAAGGTGCCCACT
TCRD-δ/D2-D3	CAAGGAAAGGGAAAAAGGAAGAA	TTGCCCCTGCAGTTTTTGTAC	ATACGCACAGTGCTACAAAACCTACAGAGACCT
TCRD-δ/D2-J1	AGCGGGTGGTGATGGCAAAGT	TTAGATGGAGGATGCCTTAACCTTA	CCCGTGTGACTGTGGAACCAAGTAAGTAACTC
TCRD-δ/V1-J1	ATGCAAAAAGTGGTCGCTATT	TTAGATGGAGGATGCCTTAACCTTA	CCCGTGTGACTGTGGAACCAAGTAAGTAACTC
TCRA-REC	AAAAAGCAACATCACTCTGTGTCT	GGCACATTAGAATCTCTCACTGA	CCAGAGGTGCGGGCCCCA
KREC-SJ	TCAGCGCCCATTACGTTTCT	GTGAGGGACACGCAGCC	CCAGCTCTTACCCTAGAGTTTCTGCACGG
KREC-CJ	CCCGATTAATGCTGCCGTAG	CCTAGGGAGCAGGGAGGCTT	AGCTGCATTTTTGCCATATCCACTATTTGGAGT

The amount of signal and coding joint of KRECs was determined by RQ-PCR as described for TRECs above. In order to calculate the change in KREC copies for each patient, the obtained Ct was compared to the Ct of his/her KREC levels before undergoing BMT using the ΛΛCt relative quantification analysis. Amplification of RNAseP (TaqMan assay, Applied Biosystems) served as a quality control to verify similar amounts of genomic DNA that were used in the assays for both the TREC and KREC analyses. Age-matched normal individuals were used as controls. Each experiment was performed in triplicate, and the threshold line for Ct determination was positioned at the same level.

### Analysis of ordered human TCR gene rearrangement

The amount of copies of various TCR gene rearrangements in a given sample was relatively quantified using RQ-PCR methodology as described above. The primers and probes are listed in Table 1. In order to calculate the fold of change in these rearrangements for each patient, the post-BMT Ct was compared to the Ct of his/her level before undergoing BMT using the ΛΛCt relative quantification analysis. Amplification of RNAseP (TaqMan assay, Applied Biosystems) was used as a quality control to verify similar amounts of genomic DNA that were used in the assays. Age-matched normal individuals were used as controls. Each experiment was performed in triplicate, and the threshold line for Ct determination was positioned at the same level.

## Results

### Patient population, characteristics and immune evaluation prior to BMT

Patients were diagnosed based on clinical findings supplemented by a basic immune workup and confirmed by genetic analysis. A total of 10 consecutive RAG-deficient SCID patients (7 males and 3 females) were included in this study (Table 2). Their age at diagnosis ranged from 1 to 6 months (mean 3±1.4 months). Seven patients had a family history suggestive of primary immunodeficiency. Three patients were diagnosed as having Omenn's syndrome. The findings of the patients' immune evaluations prior to BMT are summarized in Table 3.

**Table 2:** The clinical features of the SCID patients and their specific RAG2 genetic defect.

Patient	Age at diagnosis months	Sex	Family history	Clinical presentation	RAG2 Genetic defect
P1	3	M	**+**	SCID-Omenn	G35V
P2	3	M	**+**	SCID	G35V
P3	6	M	−	SCID	T215I
P4	3	F	+	SCID	G35V
P5	1	M	+	SCID	G35V
P6	1	F	+	SCID	T215I
P7	4	F	+	SCID-Omenn	T215I
P8	3	M	+	SCID-Omenn	G35V
P9	3	M	**−**	SCID-Omenn	G95V+E480X
P10	3	M	**−**	SCID –100% maternal engraftment	G156V

SCID – severe combined immunodeficiency.

**Table 3:** Pre-BMT immune workup of the 10 RAG2-deficient SCID patients, including flow cytometry analysis, and cell-mediated and humoral-immune evaluations.

Patient	Flow cytometry analysis of patients by lymphocytes(in 10^6^/L)						Cell mediated immunity			Humoral immunity		
	ALC(3500–9000)	CD3(1900–5900)	CD4(1400–4300)	CD8(500–1700)	CD19(600–2600)	CD56(160–950)	PHA - SI	TCR	TREC (>400)	IgG (230–1400)	IgA (0–80)	IgM (0–140)
P1	5250	4410	3675	1050	0	350	2%	UD	UD	UD	UD	UD
P2	1624	0	0	0	0	1532	1%	UD	UD	UD	UD	UD
P3	600	240	144	90	0	300	1.2%	UD	UD	33	6	9
P4	1797	29	20	7	7	1500	0.1%	clonal	UD	62	8	4
P5	221	7	6	1	1	200	0.5%	UD	UD	UD	UD	UD
P6	1190	62	46	109	0	700	6.4%	UD	UD	1120	UD	25
P7	1983	1051	892	297	0	380	ND	clonal	UD	UD	UD	UD
P8	1320	488	224	224	0	475	10.9%	clonal	UD	UD	UD	UD
P9	10686	2871	2351	855	0	3800	605%	clonal	UD	UD	UD	110
P10	5600	4612	2855	1757	1	504	3.8%	clonal	UD	433	79	UD

Control levels are presented in bracket.

ND – not done, UD- undetectable, PHA – phytohemagglutinin, SI – stimulation index (patient/control), TCR - T cell receptor, TREC - TCR excision circles (copies per 0.5 mcg DNA), ALC - absolute lymphocyte count.

### BMT procedure, complications after BMT and survival

The mean age at transplantation was 6 months (range 3–9 months) (Table 4). Six patients received an MMRD transplant using peripheral blood stem cell collection with CD34-positive selection. Three patients received MRD transplant using bone marrow and one received an MUD transplant using umbilical cord blood. There were total of 14 episodes of fever during hospitalization (Table 4). They all occurred within a few days after BMT while the patients were severely neutropenic, and all of them recovered after receiving antibiotic therapy. Six patients (60%) developed acute grade II or higher GVHD, which was reversed in all cases. As expected, patients who received an MMRD were at increased risk for developing acute GVHD. Eight patients are currently alive and have been followed-up for 6–150 months after BMT. Two patients (#1 and #2) succumbed to severe infections 22 and 36 months, respectively, after BMT. Patient #2 failed to engraft and a repeat MMRD BMT was performed 12 months after the first one. Of the 8 surviving patients, 4 (#6–8 and 10) are well and live normal lives, 3 (#3–5) still suffer from severe chronic GVHD (cGVHD), and 1 (#9) is still on prophylactic GVHD treatment with no signs of active disease. The latter patient is only 6 months after BMT and still on IVIG. The patients' clinical outcome and their basic cell -mediated and humoral-immune reconstitution after BMT in summarized in table 5.

**Table 4:** Characteristics of the BMT procedure and its related complications.

Patient	Donor	Year of BMT	Age at BMT (month)	Follow-up (months)	Conditioning	Graft	Prophylaxis	Neutrophils (days)	Acute GVHD	Febrile episodes
P1	MMRD	2005	4	36	No	PBSC/CD34	No	25	grade IV	2
P2	MMRD	20052006	47	22	No	PBSC/CD34	No	28	grade IV	2
P3	MRD	1998	7.5	146	No	BM	No	13	No	1
P4	MMRD	2002	4	92	No	PBSC/CD34	No	10	No	0
P5	MMRD	2000	1.5	132	No	PBSC/CD34	No	10	grade III	2
P6	MRD	2000	1	119	No	BM	No	11	No	1
P7	MMRD	1997	5	150	Bu Cy ATG	PBSC/CD34	CSA	8	grade III	2
P8	MRD	2008	7.5	25	Tre Flu Thi	BM	CSA+MTX	15	grade II	1
P9	MUD	2010	5	4	Bu Cy ATG	UCB	CSA	13	grade I	1
P10	MMRD	2009	5	9	Flu, Mel, Thi OKT3	PBSC/CD34	MMF	9	No	2

MRD - match related donors, MUD - match unrelated donor, MMRD - mismatch related donor, Bu – Busulfan, Cy – cyclophosphamide, ATG – anti thymocytes globulin, Tre – Treosulfan, Flu – Fludarabine, Thi – Thiotepa, Mel – melphalan, PBSC –peripheral blood stem cells, BM – Bone marrow, UCB – Umbilical cord blood, CSA – Cyclosporin, MTX – methotrexate, MMF – mycophenolate mofetil.

**Table 5:** Clinical outcome and cell -mediated and humoral-immune reconstitution after BMT in 10 RAG2-deficient SCID patients.

Patient	months post BMT	IVIG	ALC	CD3	CD4	CD8	CD56	CD20	PHA -SI	IGG	IGA	IGM	Chimerism in lymph (%)	Outcome
P1	24	yes	632	430	152	316	90	6	2.6%	486	19	27	30	Died
P2	24	yes	1840	1564	846	644	60	18	62%	ND	ND	ND	34	Died
P3	76	yes	873	306	279	105	20	61	35%	480	UD	UD	100	Alive, cGVHD
P4	72	No	2134	1643	597	1003	105	192	39%	642	118	96	50	Alive, cGVHD
P5	120	yes	2346	1947	1173	868	115	47	50%	2180	63	401	100	Alive, cGVHD
P6	6	No	1212	720	480	300	40	120	260%	ND	ND	ND	80	Alive and Well
P7	34	No	4506	3154	1262	1622	450	541	89%	ND	ND	ND	100	Alive and Well
P8	30	No	5084	3711	2695	1118	200	763	86%	1120	75	78	100	Alive and Well
P9	3	yes	747	234	112	127	350	3	ND	1110	UD	20	100	Alive and Well
P10	8	yes	2702	1973	598	1189	270	162	ND	438	UD	18	100	Alive and Well

ND-not done, UD-undetectable, PHA – phytohemagglutinin, SI – stimulation index (patient/control), ALC - absolute lymphocyte count.

### Quantification of TRECs and KRECs

**Figure 1:**
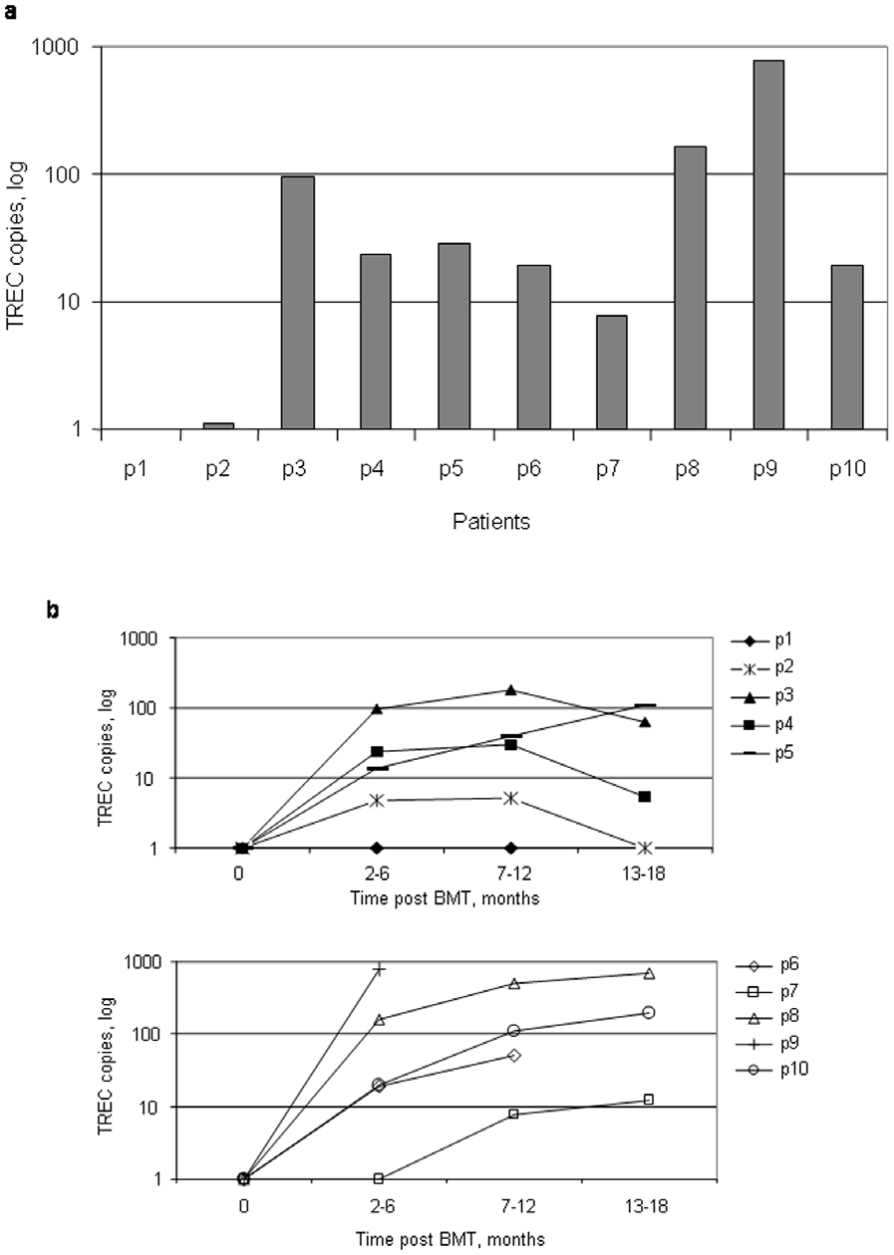
Early presence of TREC and transplant-related mortality. TREC copies were detected in all studied patients (#1–#10) at either 2–4 months (a) or various time lengths (b) post-bone marrow transplant (BMT). TREC copies were determined by real-time quantitative (RQ) PCR and calculated by comparing the obtained cycle threshold (Ct) value of a patient sample to the standard curve using an absolute quantification algorithm. Each sample was detected in triplicate.

**Figure 2:**
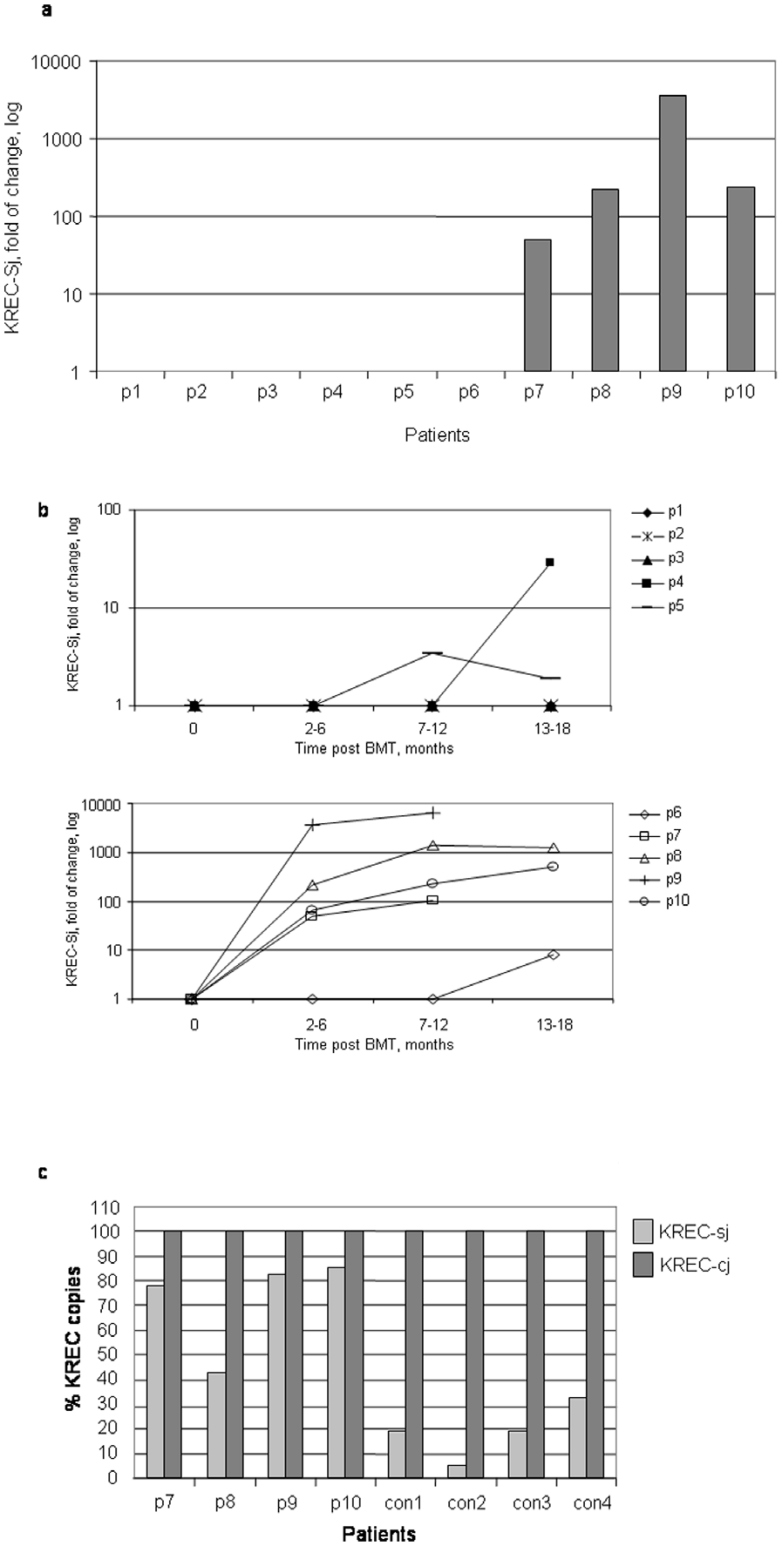
Early presence of KREC and transplant-related morbidity. KREC signal joint (sj) copies were detected in all studied patients (#1–#10) at either 2–4 months (a) or various time lengths (b) post-bone marrow transplant (BMT). KREC copies were determined by real-time quantitative (RQ) PCR and calculated by comapring the obtained cycle threshold (Ct) for each patient to the Ct of the same patient's KREC levels before undergoing BMT, using the ΛΛCt relative quantification analysis. The percent ratios between KREC-cj copies and KREC-sj copies were measured in patients with early presentation of KRECs (#7–#10) (c). Four age-matched healthy individuals were used as controls. Each sample was detected in triplicate in all experiments.

Pre-transplant TREC and KREC levels were undetectable in all patients as expected in RAG-2 SCID (data not shown). TREC and KREC copies were quantified in order to assess the neogenesis of newly produced T and B lymphocytes after BMT (Figs. 1 and 2). Early peripheral presence of newly produced T lymphocytes was observed in 8 of 10 (#3–10) patients at 2–4 months after BMT (Fig. 1a). Of them, 6 patients continued to exhibit increased thymic production of T cells over time which may reflect increased thymic function. In contrast, 4 patients showed either stable or decreased levels of TRECs (Fig. 1b). These findings were in agreement with the patients' clinical outcome. At 2–4 months after BMT, 2 patients who later displayed poor outcome (#1 and #2) had undetectable or barely detectable TREC copies (0 and 4 copies, respectively). In contrast, the 8 patients who survived the transplant (#3–10) had detectable TREC levels (ranging from 9 to 798 copies). In this latter group, no change was detected when comparing the patients who were completely well after the transplant (#6–10) with the patients who later developed transplant-related complications, mainly cGVHD (#3–5). However, the former group of patients tended to have higher levels of TREC compared to the latter group (201 vs. 48.5 average TREC copies, respectively). Uninterrupted monitoring of the TREC levels for up to 18 months after the transplant in these patients revealed gradually increased levels in all patients who remained well after the BMT (#6–10, Fig. 1b, lower panel) compared to 2 patients who developed cGVHD (#3–4, Fig. 1b, upper panel). Patients #1 and #2 continued to show undetectable or barely detectable TREC levels during 24 months of follow-up. Despite having developed cGVHD, patient #5 showed elevated levels of TREC overtime.

Early peripheral presence of newly produced B lymphocytes in the various patients was detected after BMT. KRECs were detected early in 4 of the 10 patients (#7–10) (Fig. 2a), and all of them had received pre-transplant conditioning. Three patients (#4–6) had a delayed presentation of KRECs, and three patients (#1–3) had undetectable levels of KRECs (Fig. 2b). In order to distinguish between newly produced B lymphocytes carrying signal joint episomes and replicating cells carrying only the coding joint rearrangements, measurements of these 2 distinct sequences were carried out in the patients with the early presentation of KRECs (#7–10). Most of the detected B cells in those patients had signal joint episomes (an average of 88% compared to an average of 20% that was observed in 4 aged-matched healthy controls, Fig. 2c), suggesting B cell neogenesis rather than peripheral replication in these patients. These findings were in agreement with the clinical outcome of these patients. Specifically, patients #1 and #2, who died within 24–36 months post-BMT, and patients #3, #4 and #5, who developed cGVHD, had undetectable levels of KRECs at 2–4 months after BMT, while 4 of the 5 patients who were well after BMT (#7–10) had detectable levels of KRECs at that same time point. Indeed, these patients achieved stable KREC copies during follow-up (Fig. 2b, lower panel) in contrast to patients #1–3 who continued to have undetectable KREC levels (Fig. 2b, upper panel). Two of the 3 patients with delayed presentation of KRECs (#4 and #5) developed cGVHD.

### Analysis of the T- and B-cell receptor genes

**Figure 3:**
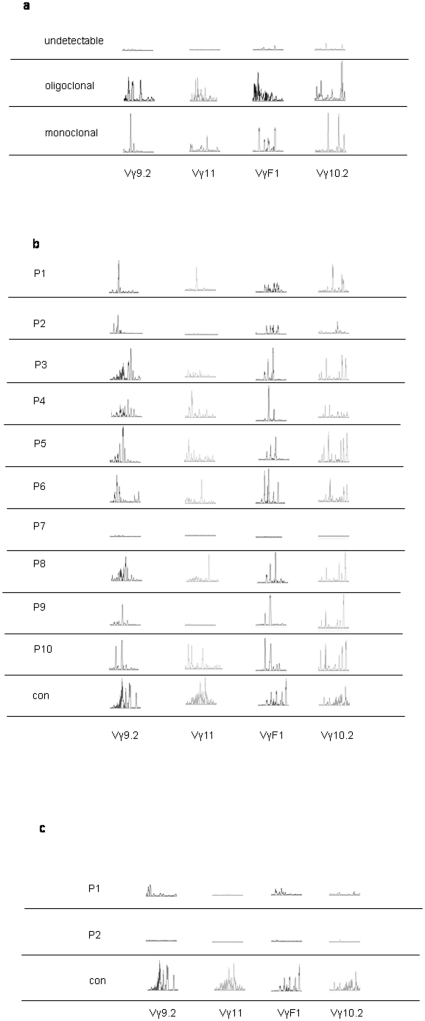
Abnormal rearrangement analysis of TCR-Vγ pre- and post-bone marrow transplant (BMT). Four different Vγ rearranged TCR genes (Vγ9.2, Vγ11, VγF1, Vγ10.2) were PCR amplified followed by Gene Scan analysis before (a) and after (b,c) BMT. A representative analysis for each pattern before transplant is presented (a). The analysis of the Vγ rearranged TCR genes in all studied patients 2–4 months post-BMT and an age-matched healthy control is shown (b). The analysis of the Vγ rearranged TCR genes in patients #1 and #2 at 4 and 16 months, respectively, before they succumbed is shown, as well as an age-matched healthy control (c).

Although it is typically not expressed on the cell surface of TCR α/β cells, the γ-chain gene remains rearranged and can be used as a marker for a proper diverse rearrangement of the TCR genes. Therefore, we performed PCR analysis of TCRv γ gene rearrangements in peripheral blood obtained from the patients before and after BMT. Analysis of 4 V γ rearranged TCR genes before BMT revealed different abnormal patterns: monoclonal in 3 patients, oligoclonal in 1 patient, and undetectable in 6 patients. These patterns correlated with the clinical phenotypes of the disease: the 4 patients who displayed the Omenn phenotype (patients #1 and #7–9) had mono- or oligoclonal patterns, while the SCID patients with no Omenn features had an undetectable repertoire. A representative repertoire is given for each pattern in figure 3a. Analysis of the 4 V γ rearranged TCR genes at 3 months after BMT showed an abnormal pattern in all patients: it was skewed in 9 patients and undetectable in 1 patient (Fig. 3b). Despite early presentation of TRECs in 8 patients (#3–10), analysis of the V γ rearranged TCR gene pattern at the same time was highly abnormal in these patients. Thus, unlike TREC evaluation, early prediction of patients' clinical outcome based on the V γ rearranged TCR gene pattern was not possible. During follow-up, 7 of the patients started to display gradual improvement in the pattern of their rearranged TCRv γ genes (data not shown). The two patients who did not survive (#1 and #2), however, had continued to demonstrate a significant abnormal TCRv γ pattern which was in correlation with their undetectable TREC levels. This abnormality was observed 4 and 16 months before they succumbed (Fig. 3c) and was attributed to their severe infection and chronic illness.

**Figure 4:**
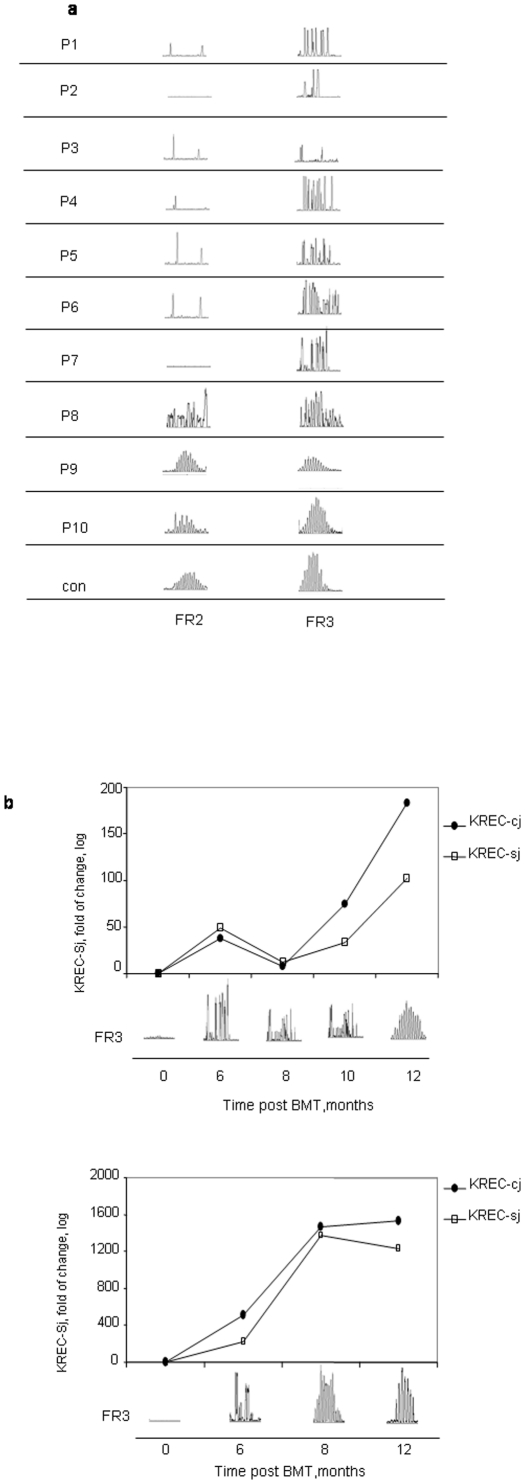
BCR rearrangement analysis and clinical outcome. Two different IgH BCR gene rearrangements (FR2 and FR3) were PCR amplified followed by Gene Scan analysis in all studied patients 2–4 months post-bone marrow transplant (BMT) and in an age-matched healthy control (a). Parallel detection of KREC levels and the FR3 IgH BCR gene rearrangement were PCR amplified, followed by RQ-PCR analysis (KREC) or by Gene Scan analysis (BCR repertoire) in patients #7 (upper panel) and #8 (lower panel) from the time of BMT and up to one year post-transplant. For KREC, both signal joint and coding joint were examined (b).

Each patient underwent PCR analysis of 2 distinct IgH BCR gene rearrangements in peripheral blood obtained from them before and after BMT. As expected, the BCR repertoire upon diagnosis of SCID was completely abnormal (data not shown). Between 2–4 months after BMT, they displayed different patterns of the BCR repertoire in agreement with their clinical outcome. Polyclonal, oligoclonal and monoclonal BCR repertoire patterns were found in 7, 1 and 2 patients, respectively, with a good outcome (#6–10) comparing to 0, 3 and 7 patients, respectively, with a poor outcome (#1–5) (Fig. 4a). Interestingly, patient #7, who had abnormal BCR gene rearrangement 3 months after BMT had a detectable KREC level at the same time. In order to test if KREC is indeed an early marker for B cell immune reconstitution, we also checked to see whether there was any correlation of the KREC level with the appearance of the BCR repertoire. As can be seen in Fig. 4b, an increased level of KREC preceded the appearance of a normal polyclonal BCR repertoire in patients #7 and #8, and most of the B cells had signal joint episomes indicative of B cell neogenesis in both of them.

### Analysis of human TCR gene rearrangement

**Figure 5:**
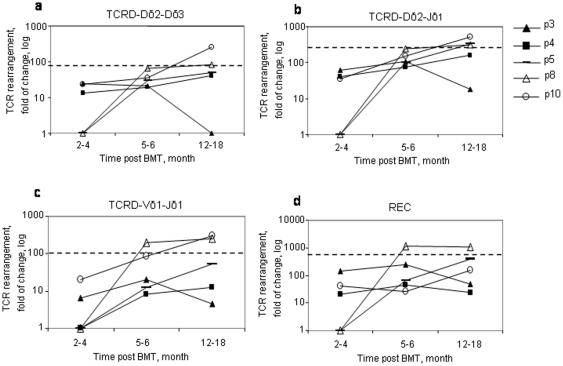
Simultaneous recovery of the ordered TCR rearrangement events and its relations to clinical outcome. Four different TCR rearrangement events (TCRD-Dδ2-Dδ3, TCRD-Dδ2-Jδ1, TCRD-Vδ1-Jδ1, TCRA-REC), indicative of different thymic maturation stages, were analyzed by real-time quantitative (RQ)-PCR overtime post transplant in 5 patients. The fold of change in these rearrangements was calculated for each patient by comparing the obtained cycle threshold (Ct) value to the Ct of his/her level before undergoing BMT, using the ΛΛCt relative quantification analysis. Three healthy controls were analyzed. Their average values are presented as a dotted line for each rearrangement event.

The TCR is created through a sequential order of recombination events between the different TCR genes (TCRD>TCRG>TCRB>TCRA). This sequential order also occurs within each individual gene and can therefore represent different stages of T cell maturation. In order to test if thymus recovery after BMT includes the simultaneous appearance of cells representing these different maturation stages, we measured 4 different TCR gene rearrangements in the peripheral blood of 5 of our RAG-2 SCID study patients over time (Fig. 5). Various rearrangements (TCRD-Dδ2-Dδ3, TCRD-Dδ2-Jδ1, TCRD-Vδ1-Jδ1, TCRA-REC) were detected 2–18 months after transplant in all of them. The amount of all these detected rearrangements increased with time in 3 patients (#5, #8 and #10) (Fig. 5a–d). In one patient (#3), however, all rearrangement events decreased over time and did so in parallel to his clinical deterioration, suggesting severe diffuse thymic suppression. Interestingly, only the latest rearrangement event (REC) decreased over time in another patient (#4, Fig. 5d), which may suggest a specific thymic impairment post-BMT rather than the general thymus suppression that had probably occurred in patient #3. Importantly, only patients with no late transplant-related complications reached normal levels of rearrangement, such as that seen in the healthy control for each event. The patients' long-term clinical outcome could be anticipated by our findings.

## Discussion

RAG-2-deficient patients have the typical T-B-SCID-variant. The undetectable TREC and KREC levels pre-transplantation common to all the patients in our cohort is clearly indicative of absent or dysfunctional T and B cells. We demonstrated that high proportions of T and B cells containing signal joints appeared early after BMT, suggesting cell neogenesis rather than cell proliferation. Their emergence was found to be closely related to the patients' clinical outcome as well as to their having undergone pre-transplant conditioning.

T cells begin to engraft quickly after hematopoietic stem cell transplantation, and substantial numbers of phenotypically mature T cells can be detected in peripheral blood within 3 months after transplantation. However, from 6 to 9 months are often required before normal numbers of circulating CD3^+^ T cells have been reached, and further characterization of these cells almost always reveals marked functional impairment and a markedly skewed TCR repertoire [13], [14]. Yet, even in the presence of considerable peripheral T cell division causing skewed TCR repertoire, TREC is present and can be detected. B cells reconstitute more slowly than T cells, and 12 to 18 months are often required to achieve relatively normal numbers of CD20^+^ cells in peripheral blood [15]. Consequently, most RAG-2-deficient SCID patients need monthly IVIG replacement therapy for a considerable period of time after BMT, until full reconstitution of B cell immunity has been achieved. Both T and B cell immune reconstitution ensure long-term survival and reduce the risk of BMT-related complications, mainly GVHD, infections and graft rejection [16]. Various studies have been performed in order to test immune reconstitution after BMT. In most of these studies, a successful immune reconstitution after transplantation was significantly associated with favorable clinical outcome [17]–[22]. Quantification of TRECs or analyses of the TCR repertoire were the most advanced assays used to determine immune reconstitution after BMT [23]. T-cell neogenesis became evident by 6 months, and normal levels of adult thymic functions were restored at 6 to 12 months after BMT [24]. Morbidity and mortality after transplantation was found in association with low TREC values [25] and BMT-related complications, such as acute GVHD or its treatment, which had a transient impact on TREC numbers [26]. In parallel, the TCR repertoire is considerably abnormal during the first year after transplantation, but it becomes normal with time, when full immune reconstitution is achieved [27]. We demonstrated peripheral presence of T cells containing signal joints early after BMT procedures, indicating that they were donor-derived naive T cells produced by the thymus, rather than proliferating cells. Truly, low TREC content reflects either reduced thymus activity or peripheral expansion [28]. However, in our case the latter is irrelevant since we show an increase in TREC content post BMT, which can be attributed only to the proliferation of new T cells produced by the thymus. In addition, TREC quantification carried out 3 months after BMT was found to be a useful tool that indicated later mortality. In general, a better estimation of thymic output eliminating factors that may affect TREC contents was suggested, combining two mathematical models. These models are based on calculating the rate of peripheral naive T cell production (using Ki67 proliferation marker), and the dynamics of TRECs [29]. A skewed TCR repertoire was still detected even when the presence of TRECs was observed shortly after BMT. We speculate that while ongoing immune responses recruited cells from the naïve T-cell pool produced by the thymus, repopulation of the T-cell compartment occurs through peripheral expansion of mature T cells secondary to antigen exposure or triggered by self-peptides. Similar to us, simultaneous quantification of TRECs and KRECs which contain the required signal joint rearrangement in patients with primary immunodeficiency after BMT was recently proposed for monitoring the appearance of newly derived thymic and bone marrow cells [30].

The different ordered TCR gene rearrangements that occur during thymic maturation of T cells represent different stages of T cell development [6]. We demonstrated that the various thymic maturation stages occur simultaneously after BMT, despite the presence of a skewed TCR repertoire. However, our patients displayed different patterns of gene rearrangement over time, a finding which may explain their clinical state during these events. As such, the amount of all TCR rearranged genes was increased in patients who had no BMT-related complications, while patients who did have BMT-related complications failed to show either general thymic suppression or late rearrangement events. The latter finding suggests a specific and late thymic T cell maturation defect. During that late stage of maturation, the AIRE protein is known to be critical in controlling thymic negative selection and ensuring that only self-tolerant T-cells are released [31]. This mechanism prevents the development of autoimmune symptoms. We and others have shown that patients who lack thymic expression of AIRE exhibit similar T cell maturation defects [32], [33]. We now speculate that the breakdown of this central tolerance mechanism may contribute to the development of late autoimmune symptoms in these patients after BMT by allowing auto-reactive clones to leak from the thymus.

B cell immune reconstitution and their early peripheral presence after BMT has not been investigated in depth. Even the decision when to discontinue gamma globulin treatment after BMT is not certain, and many parameters, including the duration of therapy, trough levels of serum IgM, IgG, or both, and/or the ability to make antigen-specific antibody after immunization are being considered. The majority of patients lacking donor B cells will require immunoglobulin replacement for life [34]. The presence of alloantibodies and high plasma B cell–activating factor levels in patients with cGVHD suggests that altered B cell homeostasis plays a role in disease pathogenesis, and that GVHD and/or its treatment hamper B-lymphopoiesis [35], [36]. It was recently used to monitor the extent of B cell immune reconstitution after BMT or enzyme replacement therapies for adenosine deaminase-deficient SCID [37]. Although B cells are known to be low in number or undetectable during the first 2 months after marrow grafting, we could detect the peripheral presence of B cells containing signal joints early after BMT. Real-time PCR detection of KRECs, despite undetectable FACS detection of B cells, g clearly indicates that measurement of KRECs is a more sensitive methodology. Furthermore, the early detection of KRECs found in some patients, could be used to anticipate their non long-term autoimmune BMT-related complications. We also observed that only those patients who received chemotherapy conditioning before BMT had early detectable KRECs. In contrast to previous assumptions that chemotherapy does not guarantee the development of that donor B cells, and that the risks associated with chemotherapy outweigh the potential for the development of B cell function, it is now widely accepted that chemotherapy administered before transplantation accelerates B cell immune reconstitution [38], as also shown by us in the current study. The issue of whether the early appearance of post-transplant B cells is due to their origin from the B cells infused with the graft or from the infused stem cells is still under debate. Since B cells containing signal joints do not duplicate during subsequent cell divisions, they represent newly produced B cells. These cells are known to be present in at least 50% of all B cells released from the bone marrow [39]. As expected, when there is a high rate of clonal and peripheral divisions in B cell malignancies, the amount of KRECs decreases proportionally [40]. Furthermore, even normal matured B cells which had already been exposed to antigenic stimulations and had undergone further divisions are known to carry smaller amounts of KRECs. Thus, although both B cell-derived and stem cell-derived B cells may theoretically coexist after grafting, we demonstrated a higher proportion of B cells containing signal joints early after BMT in patients who engrafted well and successfully survived the BMT procedure. This is indicative of early peripheral presence of B cells from their production and maturation site and not B cells that had infused with the graft. Thus, we can speculate that when engraftment is successful, peripheral B cell homeostasis had originated mainly from B cells containing signal joints rather than from matured B cell proliferation. The results of our study suggest that measurement of undetectable levels of KRECs early after BMT can be used to plan the extension of personalized GVHD prophylactic treatment in order to improve the clinical outcome of these patients. Since B cell neogenesis precedes the peripheral appearance of IgG, and assessment of host IgG production is not possible while patients are on IVIG therapy, we suggest that early detectable levels of KRECs can be used to consider shortening the duration of IVIG therapy after BMT. Moreover, given that the maintenance of immune surveillance requires the continuous supply of newly produced lymphocytes, the evaluation of KRECs offers a better marker for monitoring the efficacy of B cell compartment regeneration, as recently suggested by Serana et al [37].

The heterogeneity of our study population in terms of age at the time of transplant, the type of the donors and the conditioning protocols are possible limitations of this work. A recent report showed that the outcome after BMT for SCID is irrespective of donor choice, the conditioning regimen used, and the underlying genetic diagnosis. The overall improved survival is related to a reduced rate of infections which can be due to early diagnosis [41], or to early immune reconstitution, as we suggest. This needs to be verified by further prospective studies using similar pre-transplant parameters, given the small number of patients and the inter-patient variability of our cohort.

To summarize, a unique group of patients with profoundly depleted baseline T and B cell immunity was used to track the kinetics of T and B cell immune recovery after BMT. We showed that the early peripheral presence of newly produced B and T lymphocytes from their production and maturation sites indicated a donor stem cell origin. Early release of newly produced B and T lymphocytes was closely associated with disease outcome. Determination of immune reconstitution involves T and B cell recovery and ability to maintain immune tolerance. These capabilities will help to ensure successful engraftment, prevent infection and other BMT-related complications, and provide tolerance to self-antigens. TREC and KREC quantification are important assays for monitoring T and B cell neogenesis and homeostasis after BMT, and, as such, can serve as tools to monitor outcome and to tailor individualized therapy.
